# Neural Activity Differentiates Novel and Learned Event Boundaries

**DOI:** 10.1523/JNEUROSCI.2246-23.2024

**Published:** 2024-06-13

**Authors:** Youssef Ezzyat, Abby Clements

**Affiliations:** ^1^Department of Psychology, Wesleyan University, Middletown, Connecticut 06459; ^2^Program in Neuroscience & Behavior, Wesleyan University, Middletown, Connecticut 06459; ^3^Program in Neuroscience, Swarthmore College, Swarthmore, Pennsylvania 19081

**Keywords:** EEG, event boundaries, novelty, P300, segmentation, statistical learning

## Abstract

People parse continuous experiences at natural breakpoints called event boundaries, which is important for understanding an environment's causal structure and for responding to uncertainty within it. However, it remains unclear how different forms of uncertainty affect the parsing of continuous experiences and how such uncertainty influences the brain's processing of ongoing events. We exposed human participants of both sexes (*N* = 34) to a continuous sequence of semantically meaningless images. We generated sequences from random walks through a graph that grouped images into temporal communities. After learning, we asked participants to segment another sequence at natural breakpoints (event boundaries). Participants segmented the sequence at learned transitions between communities, as well as at novel transitions, suggesting that people can segment temporally extended experiences into events based on learned structure as well as prediction error. Greater segmentation at novel boundaries was associated with enhanced parietal scalp electroencephalography (EEG) activity between 250 and 450 ms after the stimulus onset. Multivariate classification of EEG activity showed that novel and learned boundaries evoked distinct patterns of neural activity, particularly theta band power in posterior electrodes. Learning also led to distinct neural representations for stimuli within the temporal communities, while neural activity at learned boundary nodes showed predictive evidence for the adjacent community. The data show that people segment experiences at both learned and novel boundaries and suggest that learned event boundaries trigger retrieval of information about the upcoming community that could underlie anticipation of the next event in a sequence.

## Significance Statement

People make sense of their continuous experience by segmenting it into meaningful units at event boundaries. Event boundaries influence cognitive function in a variety of ways; however, it remains unclear how different forms of uncertainty affect the parsing of continuous experiences and how such uncertainty might influence the brain's processing of ongoing events. We found that although people segment experiences at both learned and novel boundaries, brain activity diverges rapidly (250–450 ms poststimulus) in response to different types of event boundaries. The findings suggest the brain can flexibly respond to event boundaries of distinct types, which could support dynamic modulation and updating of neural activity in response to ongoing experience.

## Introduction

Time passes continuously, and yet we experience time's passage as a series of episodes that are structured around distinct and meaningful events. How does this structure arise? Research has shown that during ongoing experience, people perceive event boundaries that indicate transitions from one event to the next (Zacks et al., 2007). People parse their experiences into events in order to build and maintain mental models that enable temporal predictions about how an experience will unfold (Richmond and Zacks, 2017). Event boundaries therefore play an important role in how we experience the world and influence a variety of cognitive functions (Newtson, 1973; Zwaan, 1996; Speer and Zacks, 2005; Radvansky and Copeland, 2006; Nakano et al., 2009; Swallow et al., 2009; DuBrow and Davachi, 2014; Lositsky et al., 2016; Brunec et al., 2018; Dunsmoor et al., 2018; Heusser et al., 2018; Clewett et al., 2020; Baldwin and Kosie, 2021; Ezzyat and Davachi, 2021; Hansen et al., 2021).

Despite their wide-ranging impact and apparent importance for cognition, it remains unclear precisely how and why event boundaries are perceived during ongoing experiences. One major theory proposes that event boundaries correspond to moments of enhanced prediction error (Reynolds et al., 2007; Zacks et al., 2007; Rouhani et al., 2018; Antony et al., 2021; Ben-Yakov et al., 2022), when an internal model of the current event no longer accurately predicts one's experience (Reynolds et al., 2007; Kurby and Zacks, 2008; Richmond and Zacks, 2017). Although this model explains many of the effects of boundaries on cognition, it cannot easily account for the observation that event boundaries can occur at predictable moments of transition between two contexts (Polyn et al., 2009; Schapiro et al., 2013; Siefke et al., 2019; Sherman et al., 2023). Such predictions could emerge, for example, through learning of the statistical regularities that govern the evolution of events and experiences across time (Saffran et al., 1999; Sherman et al., 2020; Baldwin and Kosie, 2021).

Differentiating between predicted and unpredicted event boundaries could allow the brain to modulate its own response to ongoing experience. For example, event boundaries could trigger retrieval of related knowledge (Keidel et al., 2018; Cohn-Sheehy et al., 2021; Hahamy et al., 2023), especially if they are predicted, while unpredicted event boundaries could enhance attention and encoding of novel information (Den Ouden et al., 2012; Kim et al., 2017; Sinclair and Barense, 2018; Press et al., 2020; Bein et al., 2021; Chanales et al., 2021). Event boundaries that are associated with these different types of uncertainty could engage distinct neuromodulatory systems (Yu and Dayan, 2005; Poe et al., 2020) that could allow for flexible use of event boundaries to structure neural processing of ongoing experiences (Sara 2009; Zheng et al., 2022).

**Figure 1.**
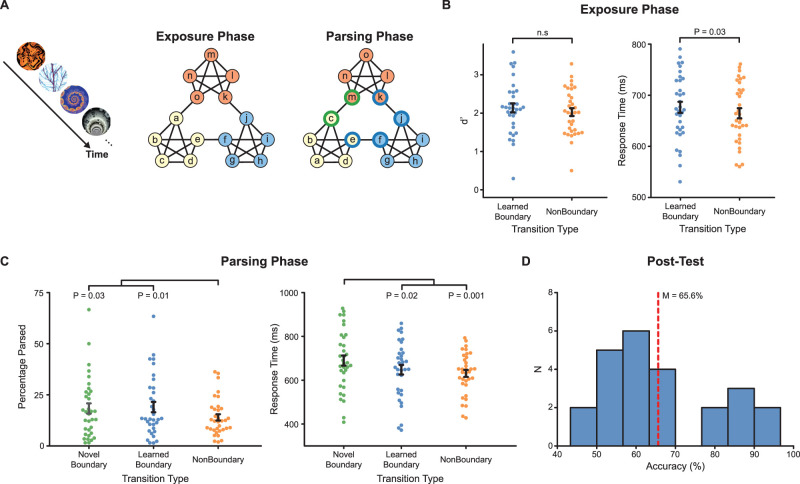
***A***, Left, Participants viewed a sequence of fractal images. Middle, During the exposure phase, transitions between fractal images were defined by an undirected graph that organized the images into communities. Right, During the parsing phase, the boundary nodes from two communities were switched with inner nodes for a block of trials, creating novel boundary transitions (green borders). Other boundary transitions remained unchanged (learned boundary, blue borders). ***B***, During the exposure phase, participants performed similarly on the rotation task (*p* = 0.53) but responded more slowly to learned boundary trials (*p* = 0.03). ***C***, During the parsing phase, nonboundary transitions were less likely to be parsed compared with novel (*p* = 0.03) and learned (*p* = 0.01) boundary transitions. Response times for novel boundary transitions were slower than for learned (*p* = 0.02) and nonboundary (*p* = 0.001) transitions. ***D***, On the posttest for explicit memory for the associations, accuracy was above chance (65.6%; *p* = 8.3 × 10^−6^).

The present study was designed to determine how people use learned and novel information to segment experiences and how neural activity differs in response to such information. Participants performed a sequence learning task (Schapiro et al., 2013; Pudhiyidath et al., 2022) in which the transitions between images in the sequence were determined by the connections in an undirected graph (Fig. 1*A*). After learning, participants again viewed a sequence of images determined by the graph structure and were asked to indicate moments in the sequence when they perceived a transition from one event to the next (i.e., event boundaries). We manipulated uncertainty by including two types of transitions between communities: novel transitions not directly possible in the learned graph and learned transitions that were possible and consistent with the initial stimulus exposure phase. We recorded scalp electroencephalography (EEG) while participants performed the task, which allowed us to investigate the neural response to event boundaries during the temporal interval of the P300, an EEG component associated with contextual novelty. Scalp EEG also allowed us to measure the timecourse over which brain activity diverges in response to novel versus learned event boundaries and to determine whether event boundaries trigger the retrieval of information that could be used to anticipate the upcoming event.

## Materials and Methods

### Participants

The 35 students from Swarthmore College (15 females; mean age, 19.1 years; range, 18–22 years) were recruited through an introductory psychology course or through an advertisement on a college Facebook group page. Participants provided informed consent in a manner approved by the Swarthmore College Institutional Review Board. Participants were reimbursed for their time either with monetary compensation ($20) or with partial credit toward their course research requirement. One participant was excluded from analysis due to technical issues with EEG data collection.

### Materials

The abstract stimuli presented to the participants were produced using the program ArtMatic Pro. Eighty-seven stimuli were created, from which seventy-four were selected based on being distinguishably different when rotated 90° (see Fig. 1*A* for example stimuli). For each participant, 15 stimuli were selected at random from the set and randomly assigned to each of the 15 nodes of the community structure graph (Fig. 1*A*).

Participants first completed the exposure phase, in which they viewed a sequence of 1,400 stimuli created by taking a random walk through the graph. Stimuli were presented one by one on the computer screen for 1.25 s each. There was no interstimulus interval, and no indication was given when the sequence moved into a new community. For the parsing phase, participants viewed a sequence of 1,200 stimuli. This sequence was created by alternating blocks of 15 stimuli generated by a pseudorandom walk through the graph and blocks of 15 items generated by a Hamiltonian path through the graph. In Hamiltonian paths, each node of the graph was visited exactly once. Each Hamiltonian path was selected pseudorandomly from a previously generated set of 15 different Hamiltonian paths, with the constraint that the path had to originate from the final node of the preceding random walk.

The purpose of including the Hamiltonian paths was twofold; first, every Hamiltonian path included at least two between-community transitions, while the 15-stimulus random walks did not necessarily include any between-community transitions; second, including the Hamiltonian paths ensured that the participants' parsing behavior could not be explained by the local statistics of the sequence, as each node was only seen once within a given Hamiltonian path. The pseudorandom walks were included to minimize unlearning of the previous temporal associations. They were pseudorandom because the program was instructed that if the walk had not moved out of a community after nine stimuli, then when the walk next landed on a boundary node, it had to move on to a different community. The alteration was made to increase the total number of community transitions within the parsing phase.

In the parsing phase, the transitions between stimuli were manipulated to introduce transitions that had not been learned during the exposure phase. The stimuli previously associated with the boundary nodes (for instance, nodes *a* and *o* on the graph in Fig. 1*A*) were swapped such that an inner node stimulus from the prior community would appear when the random walk landed on the first boundary node (*a* swapped with *b*/*c*/*d*), and an inner node stimulus from the upcoming community would appear when the random walk landed on the second boundary node (*o* swapped with *l*/*m*/*n*). These boundary transitions were impossible in the exposure phase, as an inner node from one community could not lead to an inner node from another community. Over the course of the parsing phase, we cycled which boundary nodes were manipulated, i.e., one community transition was manipulated in the first block of 200 stimuli, after which it was restored and a different community transition was manipulated and so on. The inner nodes that were swapped for the boundary nodes were randomly selected, although the selection remained constant over the 200-stimulus block. The manipulated trials made up a third of the total number of community transition trials or 4.59% of the total number of stimulus transitions.

A final posttest was given to determine participants' explicit knowledge of the community structure. The posttest involved a forced-choice paradigm where one stimulus was presented at the top of the screen and two other stimuli—a target stimulus in the same community and a lure stimulus in the other community—were presented below it. Participants had to choose which stimulus on the bottom “went with” the one on top. Each stimulus was presented four times, once with each of the other community members as the target, for a total of 60 trials.

### Procedure

Each participant was greeted and given a consent form to fill out. An EEG cap was subsequently fitted to and placed on their head. They were then shown the entire set of stimuli on the screen and told that they would be asked to decide whether each stimulus was rotated away from its initial orientation or whether it was still in its original orientation. Participants pressed one key (either J or K) when they thought the stimulus was rotated and another when it was not; key assignment was counterbalanced across participants. Reaction time data were recorded. One 500 ms beep was played if the participant answered incorrectly, and a different 500 ms beep was played if the participant failed to answer in the allotted time frame. Light foam headphones were placed over the EEG cap to allow the participants to hear the sounds. Stimuli were rotated from their initial orientation 25% of the time. The rotation task was used to keep the participants alert and attending to the stimuli; their scores from the task were calculated to ensure they had paid attention throughout the exposure phase. Every 350 stimuli or ∼7.3 min, participants were given a self-paced break. During the break, they were instructed to ring a bell, at which point the researcher would enter the testing room and adjust the impedances of the EEG cap as necessary.

The procedure for the parsing phase differed slightly for the first group of participants (*N* = 11) and the second group of participants (*N* = 24). For both groups, after the exposure phase had finished, the EEG cap was adjusted again, and the participants were told that they would now see a sequence of the same stimuli from the first part of the experiment, all in their initial orientations. For Experiment 1, participants were instructed to respond with a keypress on every trial, pressing the “K” key when they felt a “natural breakpoint or transition” had occurred in the sequence and the “J” key at all other times. Reaction time data were recorded for all trials. For Experiment 2, participants were instructed to press the spacebar when they felt a “natural breaking point or transition” had occurred in the sequence and did not respond otherwise. Reaction time data were recorded for trials during which the spacebar was pressed. For both groups, no sound feedback was given. Participants were given a self-paced break after the first 405 stimuli (8.4 min) and then after another 390 stimuli (8.1 min). During these breaks, impedances were checked again, and the caps were adjusted as needed.

After the parsing phase, the EEG caps were removed, and the participants (the second group, *N* = 24) were given the posttest. They were instructed to answer on every trial, guessing if they weren't sure. Participants were given a maximum of 30 s to answer each trial. Subsequently, the participants were asked to fill out a short survey about their experience during the study and their strategies during the parsing phase.

### EEG data acquisition and preprocessing

EEG recordings were collected using a NetAmps 300 system with a 64-channel HydroCel Geodesic Sensor Net (Electrical Geodesics). All electrodes were digitized at a sampling rate of 1,000 Hz and were referenced to electrode Cz. We used four electrodes to record the horizontal and vertical electrooculogram (EOG) of each eye. Off-line, we applied filtering to the raw EEG recordings to remove low-frequency drift (<0.5 Hz), high-frequency noise (>200 Hz), and electrical line noise (60 Hz and harmonics). We then visually inspected the voltage timeseries and manually removed intervals associated with large non-neural (e.g., muscle tension) artifacts. To remove noise related to eyeblinks and eye movements, we first decomposed the data using independent component analysis and then calculated the correlation between each independent component and the horizontal and vertical EOGs. We excluded any component with a *z*-scored correlation greater than 3 and then reconstructed the data.

We created epochs for each event type of interest by segmenting the continuous EEG recording for all electrodes using pulses that marked the onset of stimulus presentation events, which were sent from the stimulus presentation machine to the EEG acquisition machine. Epochs were defined from −100 to 1,000 ms relative to the stimulus onset. We first baseline-corrected the data for each epoch by subtracting the average voltage from the baseline period (−100 to 0 ms relative to the stimulus onset) from the entire epoch. Next, we excluded epochs with potentially nonphysiological noise using a sliding window (window length, 200 ms; step size, 50 ms) to detect peak-to-peak voltage fluctuations in excess of ±250 µV (Luck, 2014). We temporally reflected each epoch for use as mirrored pre- and postbuffer periods (Ezzyat et al., 2018; Weidemann et al., 2019) and then performed spectral decomposition using wavelets (Smith et al., 2022) with a time-bandwidth product = 4 and four cycles for each frequency. We then discarded the buffer periods. We estimated spectral power at 30 logarithmically spaced frequencies from 4 to 100 Hz (Long and Kuhl, 2019). We applied a base-10 log transform to the raw spectral power to reduce baseline differences between power at low versus high frequencies and in preparation for using parametric statistics (Cohen, 2014). We then *z*-scored the data by averaging the signal amplitude (power) within each epoch and across samples and then calculated the mean and standard deviation of the amplitude across epochs. We performed *z*-scoring separately within each channel and frequency.

### Behavioral data analysis

For the exposure phase, we used one-sample and paired *t* tests to analyze *d*′ and response times for the learned boundary and nonboundary conditions (Fig. 1). For the parsing phase, we used one-way repeated–measure ANOVAs and paired *t* tests to analyze the percentage of transitions labeled that were parsed (i.e., that participants labeled “event boundaries”) as well as response times. For the posttest data, we analyzed percent correct with a Wilcoxon signed-rank test.

### EEG data analysis

#### P300 amplitude

**Figure 2.**
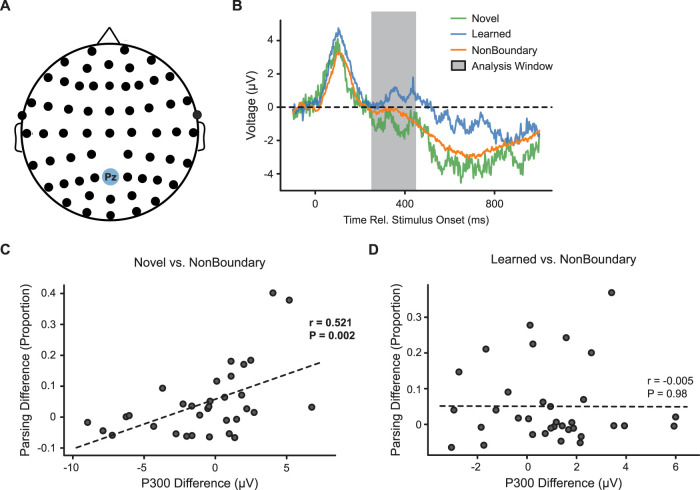
***A***, We analyzed the P300 amplitude at electrode Pz. ***B***, The average Pz amplitude differed for novel, learned, and nonboundary trials during the 250–450 ms interval poststimulus onset (*p* = 0.016). ***C***, The novel–nonboundary difference in the P300 amplitude at Pz was correlated with the novel–nonboundary difference in the probability of parsing the sequence (*p* = 0.002). ***D***, There was no correlation between P300 amplitude and parsing difference for learned versus nonboundary trials (*p* = 0.98). The correlation between P300 and parsing greater for novel boundaries than for learned boundaries (permutation test, *p* = 0.012).

For across-participant analyses of the P300 amplitude, we estimated the P300 individually for each participant by averaging the amplitude of the preprocessed voltage response between 250 and 450 ms poststimulus onset (Polich, 2012). For the analysis in Fig. 2, we calculated separate P300 amplitudes for novel boundary, learned boundary, and nonboundary trials.

#### Region of interest definition

**Figure 3.**
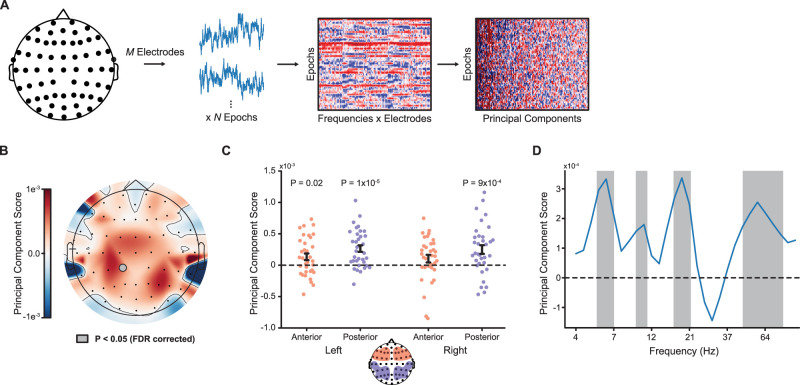
***A***, The postprocessed voltage timeseries (epochs × electrodes) was decomposed into spectral power and then averaged across time for each epoch. Using all trial types (novel/learned/nonboundary), we identified significant features using PCA applied to these frequency × electrode features. ***B***, When averaging over frequencies, only one individual electrode (CP1, gray circle) had a significant PC score. ***C***, PC scores were above zero in left anterior, left posterior, and right posterior ROIs (all *p* < 0.03 FDR-corrected). ***D***, PC scores averaged across electrodes were significantly above zero at frequencies corresponding to power in the theta, alpha, beta, and gamma bands (gray-shaded regions, *p* < 0.05 FDR-corrected).

For some analyses we grouped electrodes into four a priori regions of interest (ROIs) based broadly on their spatial locations along the left/right and anterior/posterior axes of the scalp (Weidemann et al., 2009; Long et al., 2014). Figure 3*C* shows the spatial arrangement of these ROIs.

#### Feature selection: principal component analysis

Before measuring pattern similarity within and between communities, we conducted a feature selection step by applying principal component analysis (PCA) to the spectral decomposition of the EEG timeseries (Cunningham and Yu, 2014; Lohnas et al., 2023). We applied PCA to the matrix of *z*-scored power for each epoch at each frequency × electrode (spectral power within each frequency was averaged across samples for each epoch). For features with missing data due to artifacts (see above, EEG data acquisition and preprocessing), we used a random value drawn from a Gaussian distribution with (μ,σ) equal to the mean and standard deviation of the feature across all remaining epochs (Izenman, 2008). After computing the principal component (PC) transformation, we retained components that explained a significant portion of the variance of the data (*M* = 165.4 components across participants), based on the Kaiser criterion (Manning et al., 2011). Using this subset of components, we then analyzed the relative importance of each feature by averaging the PC loadings across either frequencies or electrodes (Fig. 3*A*). We then compared the across-participant distribution of average component loadings to zero using a one-sample *t* test. We used the false discovery rate (FDR, *q *< 0.05) to correct for multiple comparisons (Benjamini and Hochberg, 1995).

#### Classification

**Figure 4.**
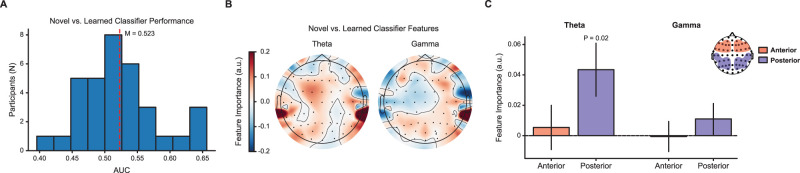
***A***, Across the group, novel versus learned boundary classification was significant (*p* = 0.03). ***B***, Topographic maps of classifier feature importance show broadly distributed differences in theta and gamma power supporting classification. ***C***, Analysis of feature importance showed that theta power in posterior electrodes contributed significantly (*p* < 0.02) to classification performance

We used multivariate classification to determine whether scalp EEG activity differed for novel compared with learned boundary transitions. We focused on the interval 250–450 ms poststimulus onset, given the prominence of the P300 component during this interval (Polich, 2012). We averaged spectral power for each epoch within this interval and then applied PCA to these frequency × electrode features. Using PCA components that explained a significant proportion of the variance in the data using the Kaiser criterion, we then used L2-penalized logistic regression to classify novel versus learned boundary epochs. To avoid overfitting the penalty parameter, we performed classification separately using eight logarithmically spaced penalty parameters between 1 × 10^−4^ and 1 × 10^4^ and averaged classifier performance across all penalty parameters. To quantify classifier performance, we used area under the receiver operating characteristic curve (AUC). We calculated AUC using leave-one-trial-out cross-validation (Hastie et al., 2009) and compared the across-participant average AUC to chance (AUC, 0.50) using a one-sample *t* test (Fig. 4*A*). We used a previously reported method to assess the relative importance of the PC features that the classifier used to discriminate novel from learned boundaries (Haufe et al., 2014). Specifically, we calculated a vector of feature importance values *A* as follows:

$$A\;=\;\frac{\Sigma_{x}\;w}{\sigma_{y}^{2}},$$

where Σ*_x_* is the data covariance matrix, *w* is the vector of classifier feature weights, and $\sigma_{y}^{2}$ is the variance of the logit-transformed classifier outputs. We then used these feature importance values to calculate a weighted average importance for the original frequency × electrode features (Fig. 4*B*,*C*).

**Figure 5.**
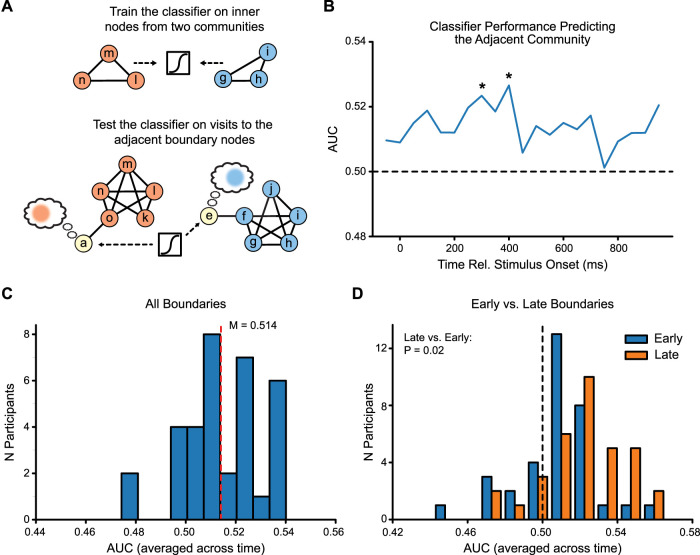
***A***, We applied classifiers to neural activity at learned boundaries to measure evidence for predictive activity for the neighboring community. ***B***, Classifier performance was above chance for much of the learned boundary presentation period (asterisks indicate *p* < 0.05 FDR-corrected time windows). ***C***, When averaged across the entire presentation period, the classifier's performance in predicting the next community (*M* = 0.514) was significant (*p* = 2 × 10^−5^). ***D***, Classifier evidence for the adjacent community was higher the more trials occurred in the same community before a visit to a boundary node (late vs early boundaries, *p* = 0.02).

For the predictive classification analyses in Figure 5, we trained classifiers to differentiate patterns of neural activity collected while viewing inner nodes from two communities (e.g., l/m/n vs g/h/i) and then applied the classifiers to adjacent boundary nodes from the held-out community (e.g., a and e; Fig. 5*A*). Classifiers were applied to learned boundary node data averaged within windows of the stimulus presentation period (window length, 100 ms; window step, 50 ms). We restricted our analyses to boundary node visits in which the same boundary node had not been visited within the previous two trials. We iterated this process across all three combinations of trained and tested communities, assessed performance using AUC for each iteration, and then averaged AUC timecourses across the three iterations of trained and tested communities (Fig. 5*B*).

#### Pattern similarity analysis

**Figure 6.**
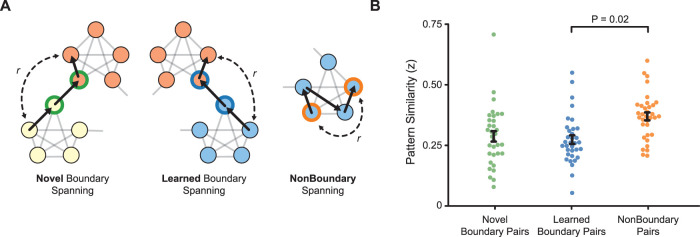
***A***, We calculated the similarity (Pearson’s *r*) between PC patterns for items that spanned novel boundaries, learned boundaries, and nonboundaries. ***B***, Pattern similarity was higher for nonboundary pairs compared with learned boundary pairs (*p* = 0.02).

After selecting significant features using PCA, we identified all pairs of inner node (i.e., not boundary nodes) epochs within Hamiltonian paths with an interitem lag of 3 for which participants did not parse the sequence. This allowed us to compare pattern similarity between pairs of items within and across communities that were matched in terms of their temporal lag and behavioral responses. The three conditions of interest are depicted in the schematic in Figure 6*A*. The novel boundary condition consisted of epoch pairs with an intervening manipulated between-community transition. The learned boundary condition consisted of epoch pairs with an intervening nonmanipulated between-community transition. The nonboundary condition consisted of the remaining epoch pairs that were separated exclusively by within-community transitions. We calculated the Pearson's correlation between each epoch pair using the significant PCs. The resulting *r* values were then transformed using the inverse hyperbolic tangent function (Fisher's *r*-to-*z* transform), averaged within each condition, and compared across participants using a one-way (novel/learned/nonboundary) repeated–measure ANOVA. We then used repeated measures *t* tests for pairwise comparisons between conditions.

#### Statistics

We report descriptive statistics as mean ± standard error of the mean. For variables assumed to be normally distributed, we used parametric inferential tests (*t* tests, ANOVA) to assess significance; otherwise, we used nonparametric tests (Wilcoxon signed-rank and permutation tests). Effect sizes are reported as Cohen's *d* or (partial) eta squared (*η*^2^).

#### Data and code availability

Data and code for this study will be available via the Open Science Framework (https://osf.io/mwjya/).

## Results

### Behavioral performance during learning

During the exposure phase, participants learned the graph structure by viewing extended sequences of the fractal images (Fig. 1*A*). Participants' task was to indicate whether the presented image was a standard or rotated version of the image. Participants performed well on the rotation task, indicating task compliance (*d*′* *= 2.03 ± 0.11; *t*_(33)_ = 19.1; *p* = 2.0 × 10^−19^; *d *= 3.28). Participants detected targets equally well for learned boundary and nonboundary transitions (learned boundary, *d*′* *= 2.14 ± 0.12; nonboundary, *d*′* *= 2.03 ± 0.10; *t*_(33)_ = 1.6; *p* = 0.12; *d *= 0.27; Fig. 1*B*). However, participants were slower to respond to learned boundary transitions compared with nonboundary transitions (learned boundary, *M* = 676 ± 11 ms; nonboundary, *M* = 665 ± 10 ms; *t*_(33)_ = 2.32; *p* = 0.03; *d *= 0.40; Fig. 1*B*). These data suggest that the exposure phase allowed participants to learn the graph structure and the differences between nodes within and between communities.

### Behavioral performance during parsing

During the parsing phase, participants were again exposed to extended sequences of fractal images and were asked to indicate with a button press when they perceived a “natural breakpoint or transition” in the sequence. As in the exposure phase, transitions from one trial to the next could be classified as either nonboundary or boundary. However, unlike the exposure phase, the parsing phase included two types of boundary transitions: learned boundaries, which had been learned previously during exposure, or novel boundaries, which were not previously encountered (Fig. 1*A*; see Material and Methods).

Based on their learning during the exposure phase, we predicted that participants would be more likely to parse the sequence at transitions between communities (learned and novel boundary), compared with transitions that remained in the same community (nonboundary). Participants' parsing behavior differed across conditions (novel, 18.3 ± 2.5%; learned, 19.0 ± 2.5%; nonboundary, 14.0 ± 1.5%; *F*_(2,66)_ = 5.17; *p* = 0.008; $\eta^{2}$ = 0.16; Fig. 1*C*, left). Compared with nonboundary transitions, participants parsed more frequently at both novel (*t*_(33)_ = 2.21; *p* = 0.03; *d *= 0.38) and learned boundary transitions (*t*_(33)_ = 2.73; *p* = 0.01; *d *= 0.47). There was no difference in parsing proportion between novel and learned boundaries (*t*_(33)_ = 0.60; *p* = 0.55). These parsing data suggest that participants perceived both novel and learned transitions as boundaries between communities.

Participant response times also differed for the three transition types (*F*_(2,66)_ = 7.59; *p* = 0.001; $\eta^{2}$ = 0.23; Fig. 1*C*, right). Here, participants were slower to parse novel boundary transitions (690 ± 23 ms) than both learned boundary (648 ± 22 ms; *t*_(33)_ = 2.44; *p* = 0.02; *d *= 0.42) and nonboundary transitions (631 ± 16 ms; *t*_(33)_ = 3.70; *p* = 0.001; *d *= 0.63). However, learned and nonboundary transitions did not differ (*t*_(33)_ = 1.27; *p* = 0.21). Taken together with the parsing data, the response time data suggest that novel boundaries were unexpected relative to learned boundaries, leading to slower responses; however, participants were still equally likely to parse the sequence at both transition types.

### Behavioral performance in the posttest

A subset of participants (*N *= 24) completed a posttest in which they were asked to pair items that they felt belonged in the same community. Participants varied in their performance on this explicit measure of association but as a group performed better than chance (*M* = 65.6 ± 3.1% vs chance = 50%; *p* = 8.3 × 10^−6^ Wilcoxon signed-rank test; *d *= 1.02; Fig. 1*D*). This suggests that participants learned the graph structure in a way that they could use to explicitly group images into communities.

To determine whether posttest performance was related to segmentation behavior, we correlated the probability of parsing the sequences at learned boundaries with overall posttest performance. We found that people who parsed the sequences more often at learned boundaries (relative to nonboundaries) also performed better on the posttest (*r*_(22)_ = 0.542; *p* = 0.006; *r*^2 ^= 0.294).

### EEG responses to between- and within-community transitions

The behavioral data from the exposure phase show that participants learned the community structure in a way that led to higher rates of parsing for boundary transitions (between communities) compared with nonboundary transitions (within a community) and above-chance explicit memory for pairwise associations between the fractal images. The behavioral data from the parsing phase showed that participants were slower to parse at novel boundaries, suggesting that although participants perceived both learned and novel boundaries as breakpoints in the sequences, novel boundaries were more unexpected relative to learned boundaries. Our first question in analyzing the EEG data was whether these behavioral differences between learned and novel boundaries were related to an established neural measure of contextual updating.

When an individual encounters information that signals contextual novelty, parietal scalp EEG recordings show a voltage deflection ∼300 ms after the stimulus onset (P300; Donchin, 1981; Donchin and Coles, 1988; Polich and Kok, 1995). In most accounts, the P300 response to contextual novelty results from differences in probability for different stimuli (Verleger, 1988); however other theoretical models propose that contextual updating need not be tied directly to differences in probability (Nieuwenhuis et al., 2005). In our task, we included event boundaries of two types: those that differed from nonboundary transitions in terms of probability (novel boundaries) and those with equivalent probabilities that nonetheless signal an update in the context (i.e., a transition to a new community; learned boundaries).

We found that EEG amplitude during the temporal window of the P300 differed across conditions (*F*_(2,64)_ = 4.44; *p* = 0.016; *η*^2 ^= 0.14). The amplitude was higher for learned boundary transitions compared with that for nonboundary transitions (*t*_(33)_ = 2.45; *p* = 0.02; *d *= 0.43), consistent with the idea that moving between communities leads to contextual updating associated with the P300. Learned boundary transitions were also higher than novel boundary transitions (*t*_(32)_ = 2.63; *p* = 0.01; *d *= 0.47), which did not differ in the overall amplitude from nonboundary transitions (*t*_(32)_ = −1.08; *p* = 0.29). As a comparison, we also analyzed the EEG amplitude for the early peak that was evident between 50 and 250 ms relative to the stimulus onset. However, there were no differences in the amplitude during this early window (*F*_(2,64)_ = 1.53; *p* = 0.22).

Next, we asked how the P300 amplitude was related to participants' behavioral segmentation of the sequence into events. If the P300 reflects updating of a neural context at event boundaries, we predicted that the size of the P300 response should correlate with the probability of parsing the sequence at an event boundary. Consistent with our hypothesis, we found that the increased P300 amplitude for novel boundaries compared with nonboundaries at electrode Pz was correlated with increased probability of parsing the sequence at novel boundaries (Fig. 2*B*; *r*_(31)_ = 0.521; *p* = 0.002; *r*^2 ^= 0.271). In contrast, there was no such correlation when comparing learned boundaries and nonboundaries (Fig. 2*C*; *r*_(32)_ = −0.005; *p* = 0.98). The novel boundary correlation was significantly greater than the correlation for learned boundaries (permutation test, *p* = 0.012). Together, these analyses suggest that the P300 amplitude is sensitive to transitions between events, related to explicit event segmentation for novel boundaries, and implicit detection of a new context for learned boundaries.

### PCA of spectral features

Having shown that a larger parietal P300 response to novel boundaries occurs alongside a higher probability of parsing the sequence at such boundaries (but not learned boundaries), our subsequent analyses focused on understanding the spatial and temporal distribution of EEG responses to novel and learned boundaries. Before doing so, we first sought to identify and characterize the latent features that explained significant variance in the data using PCA (Manning et al., 2011; Cunningham and Yu, 2014; Lohnas et al., 2023). We applied spectral decomposition to the voltage data from the image presentation epochs for all stimuli and retained significant PCs based on the Kaiser criterion (Fig. 3*A*; see Materials and Methods).

We first asked what PCA-identified features explained significant variance across participants. We began by mapping the across-scalp topography of individual electrodes with PC weights that were reliably different from zero. Larger weights for some electrodes and not others would suggest that some scalp locations contributed more strongly than others to the latent PC features in our data. We identified only one electrode (CP1) with a significant PC component score (Fig. 3*B*, filled gray circle), suggesting that, in general, multivariate patterns of activity in our task were not dominated by the responses of individual electrodes but instead broadly distributed across space. However, when aggregating electrodes into left/right hemispheres × anterior/posterior locations, PC component scores were higher in posterior regions (*F*_(1,33)_ = 4.71; *p* = 0.04; $\eta_{p}^{2}$ = 0.22; Fig. 3*C*). There was no difference between the left and right hemispheres (*F*_(1,33)_ = 0.11; *p* = 0.74) and no interaction (*F*_(1,33)_ = 0.02; *p* = 0.89). PC scores were greater than zero in the left anterior (*t*_(33)_ = 2.53; *p* = 0.02; *d *= 0.44), left posterior (*t*_(33)_ = 5.14; *p* = 1.2 × 10^−5^; *d *= 0.88), and right posterior regions (*t*_(33)_ = 3.63; *p* = 0.001; *d *= 0.62; Fig. 3*C*).

We next asked whether PC scores indicated that particular frequency bands explained significant variance in the data or instead whether the PCs loaded more uniformly across the frequency spectrum. Here, we found that PCs loaded more strongly on specific frequency bands: 5–7 Hz (theta), 9–10 Hz (alpha), 17–21 Hz (beta), and 45–80 Hz (gamma; Fig. 3*D*, gray-shaded regions; *p* < 0.05 FDR-corrected). There were no frequencies at which the PC scores were significantly below zero after FDR correction, at least when averaged across all electrodes. Component scores were also similar when comparing the significant theta, alpha, beta, and gamma bands to each other (*F*_(3,99)_ = 1.02; *p* = 0.39). Taken together with the electrode-level analysis, the PCA analysis suggests there is significant variability in spectral activity in our task; the data also suggest these latent features are distributed widely across both space and frequency.

### Neural representation of novel and learned boundaries

Having established and characterized latent spectral features in the data using PCA, we next asked whether these features reflect differences in neural activity in response to novel and learned boundaries. To answer this question, we used the PC features to train a classifier (L2-penalized logistic regression; see Materials and Methods) to differentiate neural activity for novel versus learned boundary trials. Classification was significant across the group (mean cross-validated AUC, 0.523; *t*_(33)_ = 2.25; *p* = 0.03; *d *= 0.39; Fig. 4*A*), showing that the neural response to novel and learned boundaries can be decoded from multivariate features broadly distributed across space and frequency. In contrast, classification was not greater than chance when using only the Pz amplitude (AUC, 0.507; *p* = 0.43); the difference between the multivariate and univariate classifiers was not significant (*p* = 0.16). This suggests that group-level differences in the amplitude at electrode Pz (i.e., a univariate classifier) are not sufficient to differentiate novel versus learned boundaries at the individual trial level.

To determine what features were important to the classifier's decisions, we analyzed the average classifier feature importance estimates (Haufe et al., 2014). Specifically, we compared the scalp topography of classifier feature importance estimates separately for theta and gamma frequencies (Fig. 4*B*). We focused on the theta and gamma bands based on prior work indicating these frequencies are broadly associated with successful performance across a range of cognitive states (Klimesch, 1999; von Stein and Sarnthein, 2000; Burke et al., 2013). Theta and gamma activities also change in response to neuromodulatory systems that are associated with environmental uncertainty (Berridge and Foote, 1991; Sara, 2015; Mather et al., 2016). The maps suggested that the classifier made use of broadly distributed differences in both theta and gamma power. To determine whether individual ROIs or frequencies were especially important for classification, we aggregated the classifier feature importance estimates separately for features in the theta versus gamma bands, as well as posterior versus anterior electrodes. Feature importance was higher for posterior compared with anterior ROIs (*F*_(1,33)_ = 4.45; *p* = 0.04; $\eta_{p}^{2}$ = 0.07; Fig. 4*C*); this effect was driven by increased feature importance in the theta band (*t*_(33)_ = 2.46; *p* = 0.02; *d *= 0.42).

### Predictive representations at community boundaries

In our task, novel and learned boundaries both indicate a transition to a new community; however, they differ critically in whether or not this transition is predictable based on prior learning. We therefore asked whether neural activity before a transition reflects learned predictions about what should happen next in the sequence (Sherman and Turk-Browne, 2020; Lee et al., 2021). Although prior fMRI evidence has identified predictive representations following learning of sequential statistical regularities (Schapiro et al., 2012; Hindy et al., 2016), little is known about the temporal evolution of predictive responses and how it relates to potential upcoming events. We sought to address this question with our data, taking advantage of the temporal resolution of the EEG signal. To identify predictive responses to a stimulus, we trained multivariate classifiers on neural activity for inner node trials for a given community and then tested the trained classifier on activity recorded during visits to the adjacent boundary nodes (Fig. 5*A*).

If boundary nodes carry information that predicts the upcoming community, then adjacent-community classification should be significant during viewing of the boundary nodes. Further, if predictive responses reflect the retrieval of a distinct representation of the adjacent community, the timecourse of classification should track the timecourse of retrieval of this association (Staresina et al., 2012). We trained and tested classifiers separately for time windows across the epoch, taking advantage of the temporal resolution of our data to determine the timecourse over which predictive information emerges during boundary node viewing. We found that classifier performance was significant between 250 and450 ms relative to the stimulus onset (Fig. 5*B*, asterisks indicate FDR-corrected time windows). Classifier performance was also significant when averaging across the entire stimulus presentation interval (*M* = 0.514; *p* = 2 × 10^−5^; *d *= 0.85; Fig. 5*C*). In contrast, when using classifiers trained on permuted category labels, performance was significantly lower than when using the true labels (*t*_(33)_ = 4.08; *p* = 3 × 10^−4^; *d *= 0.71) and no different from chance in any time window nor when averaged across the stimulus presentation interval (*p* = 0.98).

The preceding data suggest that a predictive response emerges ∼250 ms after the stimulus onset, consistent with the retrieval of associated information about the adjacent community during the temporal interval of the P300. We next asked whether spending more time in a community before visiting a boundary node led to stronger predictions for the adjacent community. To answer this question, we applied the classifiers to boundary nodes, splitting trials into early and late groups based on the median number of trials spent in one community before moving to a different community. Classification was higher for late boundaries (late AUC, 0.521 ± 0.004) compared with that for early boundaries (early AUC, 0.509 ± 0.004; *t*_(33)_ = 2.42; *p* = 0.02; *d *= 0.42; Fig. 5*D*), suggesting that neural predictions at boundaries are stronger when more time has already been spent within a given community.

The preceding analyses suggest that community boundaries evoke neural activity that predicts the adjacent community. Because we trained our classifiers only on inner node trials, it is unclear whether these predictions reflect general information about the adjacent community or specific information about upcoming nodes. For example, when visiting node *a*, is there specific evidence for node *o* beyond general evidence node *o*'s community? To test this, we took advantage of an aspect of our design, which is that some inner node trials were swapped with boundary nodes from the same community for portions of the session, thus temporarily placing inner nodes in “boundary” positions (i.e., the novel boundaries). We asked whether pattern similarity between *a* and *o* (learned–learned pair) differed from pattern similarity between *a* and *m* (learned–novel pair). If the predictive activity at boundaries predominantly reflects item-level representations, then learned–learned pairs should be more similar than learned–novel pairs. If instead the activity reflects community-level representations, then there should be no difference. Consistent with a general prediction for the upcoming community, we found that learned–learned pattern similarity was not different from learned–novel pattern similarity (0.394 vs 0.392; *t*_(33)_ = 0.28; *p* = 0.78).

The previous analyses suggest that a reliable predictive response emerges ∼250 ms after the stimulus onset, consistent with the retrieval of associated information about the adjacent community during the temporal interval of the P300. However, it is also possible that significant classification reflects a more general process that is not specific to boundary nodes. To test whether boundary trials are unique in this way, we took the classifiers from the preceding analysis (trained on inner nodes from two communities) and tested them on inner nodes from the held-out community that were visited just prior to a boundary node (preboundary nodes). If above-chance classification is due to evidence about the adjacent community that is unique to boundaries, then applying the classifiers to preboundary nodes should lead to chance performance. Indeed, we found that classification of preboundary nodes was not significantly different from chance for any individual time window (all FDR-corrected *p* values, >0.77) nor when averaging across the stimulus presentation epoch (*M* = 0.50; *p* = 0.18). In addition, classification of boundary nodes was significantly higher than classification of preboundary nodes (*t*_(33)_ = 3.00; *p* = 0.005; *d* = 0.52). Taken together, these data suggest boundary trials contain unique information about the adjacent community.

### Neural representation of community structure

Given that a representation of the adjacent community emerges 250–450 ms after encountering a boundary stimulus, we next asked whether learning of the community in our task was reflected in the neural representations of inner nodes (Schapiro et al., 2013). To determine whether learning influenced the pattern of activity recorded at the scalp, we used the PC features to calculate the correlation between pairs of items that spanned novel boundaries, learned boundaries, and nonboundaries (Fig. 6*A*). We predicted that learning the community structure during the exposure phase would lead to higher pattern similarity in the parsing phase for nonboundary spanning pairs (Schapiro et al., 2013; Pudhiyidath et al., 2022). We found higher similarity for pairs from the same community compared with those from different communities (nonboundary, 0.35 ± 0.03 vs learned boundary = 0.29 ± 0.02; *t*_(31)_ = 2.51; *p* = 0.02; one-way ANOVA: *F*_(2,62)_ = 3.42; *p* = 0.04; $\eta_{p}^{2}$ = 0.11; Fig. 6*B*). Nonboundary pattern similarity did not differ when compared with pairs that were separated by a manipulated community transition (novel boundary, 0.31 ± 0.02; *t*_(31)_ = 1.36; *p* = 0.19). There was also no difference in pattern similarity between pairs that spanned learned and novel community boundaries (*t*_(31)_ = 1.38; *p* > 0.18). These findings show that learned community structure shapes the neural representations of events as measured with scalp EEG: for the two conditions that were consistent with prior learning (nonboundary and learned boundary), we found greater similarity for items within a community compared with those across communities.

Finally, we also asked whether the pattern similarity effects were related to posttest performance. However, greater nonboundary similarity (relative to learned boundaries) was not correlated with posttest performance (*r*_(21)_ = 0.062; *p* = 0.78).

## Discussion

We used a sequential image presentation paradigm combined with scalp EEG to measure neural responses to event boundaries that were either consistent or inconsistent with previous learning. We found that participants parsed the sequences at novel and learned boundaries but were slower to do so for novel boundaries, suggesting a violation of sequential expectations. Segmentation at novel boundaries was also associated with a scalp P300 response. Novel and learned boundaries produced distinct patterns of neural activity across the scalp, and activity during learned boundaries suggested prediction of the upcoming community. The findings suggest the brain can flexibly respond to event boundaries of distinct types, which could support dynamic modulation and updating of neural activity in response to ongoing experience.

Our study provides the first evidence linking EEG activity during the window of the P300 with information processing at event boundaries. The P300 typically occurs between 250 and 450 ms after a stimulus and is thought to reflect updating of an internal context representation in response to salient sensory information in the environment (Donchin and Coles, 1988). Theoretical accounts have suggested that the P300 scalp response may reflect arousal-mediated locus ceruleus release of norepinephrine (Pineda et al., 1989; Nieuwenhuis et al., 2005), consistent with the hypothesized role of norepinephrine in resetting neural network activity (Bouret and Sara, 2005; Sara, 2009). Based on these models, we predicted that P300 activity would differ for novel compared with learned event boundaries if these two types of boundaries differentially engaged the locus ceruleus arousal system. Consistent with this prediction, we found that multivariate classifiers reliably discriminated between novel and learned event boundaries during the P300 temporal window. In addition, we found that the parietal P300 amplitude itself correlates with the probability that people perceive event boundaries at novel boundary transitions. Our data therefore identify a functional role for P300-related scalp EEG activity in processing and perceiving event boundaries and suggest that locus ceruleus norepinephrine activity may play a key role in the brain's response to event boundaries. In this way, our data complement previous empirical (Clewett et al., 2020) and theoretical work (Swallow et al., 2022) that relate the brain's arousal system and perception of event boundaries.

We identified a role for low-frequency spectral features (in the theta range) at posterior electrodes in both our PCA (Fig. 3*C*,*D*) and our classification of novel versus learned boundaries (Fig. 4*B,C*). Topographic maps from both analyses suggested the theta effects were strongest at lateral and inferior electrodes spanning the temporal and parietal lobes, consistent with previous work that has identified the temporoparietal junction as a key cortical generator of the scalp P300 (Knight et al., 1989; Yamaguchi and Knight, 1991; Verleger et al., 1994). The temporoparietal junction is a core area of the default network (Andrews-Hanna et al., 2010), and activity in this network supports people's ability to understand sequences of events in continuous experience through integration of information across varying timescales (Simony et al., 2016). Other research has suggested that theta activity reflects neural network synchronization that supports the organization of information in working memory (Klimesch, 1999; Sauseng et al., 2010). In the context of our task, working memory representations related to a representation of the current event may undergo differential updating at novel versus learned between-community transitions. Thus, while speculative, our data are consistent with a role of temporoparietal theta activity in the updating of event representations at transitions between ongoing events.

By first exposing participants to an extended sequence of semantically meaningless stimuli, we controlled how participants learned to segment the stimuli into groups. In everyday human experience, we segment events based on a wealth of prior knowledge about how events relate to each other and about causal relations between different events, antecedents, and outcomes (Zacks and Tversky, 2001). Because we controlled the background knowledge that participants learned in our task, we were able to precisely manipulate whether and in what way a presented stimulus was consistent or inconsistent with previous learning. However, it remains an open question whether or not human semantic knowledge generates expectations that are similar to those that could be generated from the learning in our task. Causal inference is likely to be a key driver of event segmentation in naturalistic settings (Gershman and Niv, 2010; Franklin et al., 2020; Shin and DuBrow, 2021), which may arise from semantic knowledge and guide or constrain how organisms generate expectations in these settings.

Previous research has shown that people can use statistical learning to rapidly extract information about structure at multiple scales in the environment (Schapiro et al., 2016; Forest et al., 2022), which can support processes such as categorization and inference (Schlichting and Preston, 2015; Morton et al., 2020; Pudhiyidath et al., 2022). A key question in this domain concerns the nature of the learned representation(s) that guide behavior (Mack et al., 2020; Zeithamova and Bowman, 2020). After the learning phase of our task, people could conceivably represent the specific item–item transitions encountered during learning or general knowledge of each item's community membership in the absence of information about specific transitions. If people represent specific item transitions, then they should be sensitive to violations of this learning, as reflected in the current study by novel boundary transitions. Alternatively, if people represent only generalized knowledge about the community structure, their performance should not differ for novel compared with learned boundary transitions, since in both cases there is a sequence transition between communities. We found evidence consistent with both types of representation. Participants were equally likely to parse transitions at novel and learned boundaries, consistent with an abstract and general representation about each item's community membership. At the same time, participants were also slower to parse novel compared with learned transitions, suggesting they were also sensitive to the novel item–item transition. Thus, participants seem to represent structure in our task at multiple scales, consistent with recent theoretical accounts proposing a mixture of representations supporting decision-making and prediction about events after learning (Momennejad et al., 2017; Franklin et al., 2020).

The ability to predict the future state of the environment is a fundamental goal of neural information processing (Rao and Ballard, 1999; Friston, 2005; Hutchinson and Barrett, 2019) and is thought to be one of the primary reasons why people segment their experiences into events (Richmond and Zacks, 2017; Stawarczyk et al., 2021). Prediction error plays a critical role in this process, allowing people to update their event-based predictions in response to inputs that violate expectations (Reynolds et al., 2007; Zacks et al., 2007, 2011). However, previous research has also shown that prediction errors per se are not always necessary for segmentation; for example, people can segment experiences based on shared temporal context (Schapiro et al., 2013) or changes in predictive uncertainty (Hansen et al., 2021). Theoretically, outcomes that are associated with greater uncertainty provide more information than outcomes associated with less uncertainty (Shannon, 1948). When considered along with our observation of distinct electrophysiological responses to novel and learned boundaries, the data suggest a link between the anticipation of and response to event boundaries and processing of ongoing experience (Baldwin and Kosie, 2021).

Previous work has suggested that event boundaries may be advantageous moments for the brain to encode an episodic memory for a just-experienced event (Ben-Yakov and Dudai, 2011; Ben-Yakov et al., 2013; Baldassano et al., 2017; Michelmann et al., 2021; Lu et al., 2022) via rapid reinstatement of a representation of the event (Clewett and Davachi, 2017; Griffiths and Fuentemilla, 2020). Consistent with this hypothesis, scalp EEG signals at event boundaries are more similar to recently encountered sequences when the items in the sequence are trial-unique (and putatively episodic) as opposed to repeated across trials (Sols et al., 2017). This pattern similarity correlates with later memory performance, suggesting a link between neural reinstatement at event boundaries and memory. Scalp EEG pattern similarity between event boundaries and preceding events is similarly enhanced and related to memory performance under naturalistic stimulus conditions (Silva et al., 2019). In our data, we find distinct electrophysiological representations of event boundaries when they arise under different forms of uncertainty (novel vs learned boundary classification; Fig. 4*A*), which suggests a potential means for the brain to shape this boundary-related retrieval process toward events at different scales (Duncan and Schlichting, 2018; Cohn-Sheehy et al., 2022; Pu et al., 2022; Hahamy et al., 2023) or even to bias the memory system away from retrieval of the preceding event and toward encoding of new information (Heusser et al., 2018; Turker and Swallow, 2019; Brunec et al., 2020; Rouhani et al., 2020; Smith et al., 2022).

In sum, we observed behavioral and electrophysiological differences in how people process type of event boundaries after learning. Scalp EEG recordings showed rapid divergence in response to event boundaries that differed in their consistency with prior learning and in a way that suggested a role for neuromodulator-mediated arousal systems. Our study advances our understanding of the mechanisms by which the human brain organizes continuous experience into distinct and meaningful events.

## References

[B1] Andrews-Hanna JR, Reidler JS, Sepulcre J, Poulin R, Buckner RL (2010) Functional-anatomic fractionation of the brain’s default network. Neuron 65:550–562. 10.1016/j.neuron.2010.02.005 20188659 PMC2848443

[B2] Antony JW, Hartshorne TH, Pomeroy K, Gureckis TM, Hasson U, McDougle SD, Norman KA (2021) Behavioral, physiological, and neural signatures of surprise during naturalistic sports viewing. Neuron 109:377–390. 10.1016/j.neuron.2020.10.029 33242421

[B3] Baldassano C, Chen J, Zadbood A, Pillow JW, Hasson U, Norman KA (2017) Discovering event structure in continuous narrative perception and memory. Neuron 95:709–721. 10.1016/j.neuron.2017.06.041 28772125 PMC5558154

[B4] Baldwin DA, Kosie JE (2021) How does the mind render streaming experience as events? Top Cogn Sci 13:79–105. 10.1111/tops.12502 32529736

[B5] Bein O, Plotkin NA, Davachi L (2021) Mnemonic prediction errors promote detailed memories. Learn Mem 28:422–434. 10.1101/lm.053410.121 34663695 PMC8525423

[B5a] Benjamini Y, Hochberg Y (1995) Controlling the false discovery rate: a practical and powerful approach to multiple testing. J R Stat Soc Ser B Methodol 57:289–300. 10.1111/j.2517-6161.1995.tb02031.x

[B6] Ben-Yakov A, Dudai Y (2011) Constructing realistic engrams: poststimulus activity of hippocampus and dorsal striatum predicts subsequent episodic memory. J Neurosci 31:9032–9042. 10.1523/JNEUROSCI.0702-11.2011 21677186 PMC6622928

[B7] Ben-Yakov A, Eshel N, Dudai Y (2013) Hippocampal immediate poststimulus activity in the encoding of consecutive naturalistic episodes. J Exp Psychol Gen 142:1255. 10.1037/a0033558 23815458

[B8] Ben-Yakov A, Smith V, Henson R (2022) The limited reach of surprise: evidence against effects of surprise on memory for preceding elements of an event. Psychon Bull Rev 29:1053–1064. 10.3758/s13423-021-01954-5 34173187 PMC9166837

[B9] Berridge CW, Foote SL (1991) Effects of locus coeruleus activation on electroencephalographic activity in neocortex and hippocampus. J Neurosci 11:3135–3145. 10.1523/JNEUROSCI.11-10-03135.1991 1682425 PMC3058938

[B10] Bouret S, Sara SJ (2005) Network reset: a simplified overarching theory of locus coeruleus noradrenaline function. Trends Neurosci 28:574–582. 10.1016/j.tins.2005.09.002 16165227

[B11] Brunec IK, Moscovitch M, Barense MD (2018) Boundaries shape cognitive representations of spaces and events. Trends Cogn Sci 22:637–650. 10.1016/j.tics.2018.03.013 29706557

[B12a] Brunec IK, Robin J, Olsen RK, Moscovitch M, Barense MD (2020) Integration and differentiation of hippocampal memory traces. Neurosci Biobehav Rev 118:196–208. 10.1016/j.neubiorev.2020.07.024 32712280

[B12] Burke JF, Zaghloul KA, Jacobs J, Williams RB, Sperling MR, Sharan AD, Kahana MJ (2013) Synchronous and asynchronous theta and gamma activity during episodic memory formation. J Neurosci 33:292–304. 10.1523/JNEUROSCI.2057-12.2013 23283342 PMC3711714

[B13] Chanales AJ, Tremblay-McGaw AG, Drascher ML, Kuhl BA (2021) Adaptive repulsion of long-term memory representations is triggered by event similarity. Psychol Sci 32:705–720. 10.1177/0956797620972490 33882251 PMC8726589

[B13a] Clewett D, Davachi L (2017) The ebb and flow of experience determines the temporal structure of memory. Curr Opin Behav Sci 17:186–193. 10.1016/j.cobeha.2017.08.013 29276730 PMC5739077

[B14] Clewett D, Gasser C, Davachi L (2020) Pupil-linked arousal signals track the temporal organization of events in memory. Nat Commun 11:4007. 10.1038/s41467-020-17851-9 32782282 PMC7421896

[B15] Cohen MX (2014) Analyzing neural time series data: theory and practice. Cambridge, MA: MIT press.

[B16] Cohn-Sheehy BI, Delarazan AI, Reagh ZM, Crivelli-Decker JE, Kim K, Barnett AJ, Zacks JM, Ranganath C (2021) The hippocampus constructs narrative memories across distant events. Curr Biol 31:4935–4945. 10.1016/j.cub.2021.09.013 34592172 PMC9373723

[B16a] Cohn-Sheehy BI, Delarazan AI, Crivelli-Decker JE, Reagh ZM, Mundada NS, Yonelinas AP, Zacks JM, Ranganath C (2022) Narratives bridge the divide between distant events in episodic memory. Mem Cognit 50:478–494. 10.3758/s13421-021-01178-x 33904017 PMC8546012

[B17] Cunningham JP, Yu BM (2014) Dimensionality reduction for large-scale neural recordings. Nat Neurosci 17:1500–1509. 10.1038/nn.3776 25151264 PMC4433019

[B18] Den Ouden HE, Kok P, De Lange FP (2012) How prediction errors shape perception, attention, and motivation. Front Psychol 3:548. 10.3389/fpsyg.2012.00548 23248610 PMC3518876

[B18a] Donchin E (1981) Presidential address, 1980. Surprise!...Surprise? Psychophysiology. 18:493–513. 10.1111/j.1469-8986.1981.tb01815.x 7280146

[B19] Donchin E, Coles MG (1988) Is the P300 component a manifestation of context updating? Behav Brain Sci 11:357–374. 10.1017/S0140525X00058027

[B20] DuBrow S, Davachi L (2014) Temporal memory is shaped by encoding stability and intervening item reactivation. J Neurosci 34:13998–14005. 10.1523/JNEUROSCI.2535-14.2014 25319696 PMC4198540

[B20a] Duncan KD, Schlichting ML (2018) Hippocampal representations as a function of time, subregion, and brain state. Neurobiol Learn Mem 153:40–56. 10.1016/j.nlm.2018.03.006 29535044

[B21] Dunsmoor JE, Kroes MC, Moscatelli CM, Evans MD, Davachi L, Phelps EA (2018) Event segmentation protects emotional memories from competing experiences encoded close in time. Nat Hum Behav 2:291–299. 10.1038/s41562-018-0317-4 30221203 PMC6136428

[B22] Ezzyat Y, et al. (2018) Closed-loop stimulation of temporal cortex rescues functional networks and improves memory. Nat Commun 9:365. 10.1038/s41467-017-02753-0 29410414 PMC5802791

[B23] Ezzyat Y, Davachi L (2021) Neural evidence for representational persistence within events. J Neurosci 41:7909–7920. 10.1523/JNEUROSCI.0073-21.2021 34330773 PMC8445049

[B23a] Franklin NT, Norman KA, Ranganath C, Zacks JM, Gershman SJ (2020) Structured event memory: a neuro–symbolic model of event cognition. Psychol Rev 127:327–361. 10.1037/rev0000177 32223284

[B23b] Forest TA, Finn AS, Schlichting ML (2022) General precedes specific in memory representations for structured experience. J Exp Psychol Gen 151:837–851. 10.1037/xge0001104 34780215

[B24] Friston K (2005) A theory of cortical responses. Philos Trans R Soc Lond B Biol Sci 360:815–836. 10.1098/rstb.2005.1622 15937014 PMC1569488

[B25] Gershman SJ, Niv Y (2010) Learning latent structure: carving nature at its joints. Curr Opin Neurobiol 20:251–256. 10.1016/j.conb.2010.02.008 20227271 PMC2862793

[B26] Griffiths BJ, Fuentemilla L (2020) Event conjunction: how the hippocampus integrates episodic memories across event boundaries. Hippocampus 30:162–171. 10.1002/hipo.23161 31566860

[B27] Hahamy A, Dubossarsky H, Behrens TE (2023) The human brain reactivates context-specific past information at event boundaries of naturalistic experiences. Nat Neurosci 26:1–10. 10.1038/s41593-023-01331-6 37248340 PMC7614642

[B28] Hansen NC, Kragness HE, Vuust P, Trainor L, Pearce MT (2021) Predictive uncertainty underlies auditory boundary perception. Psychol Sci 32:1416–1425. 10.1177/0956797621997349 34409898

[B28a] Hastie T, Tibshirani R, Friedman JH, Friedman JH (2009) The elements of statistical learning: data mining, inference, and prediction, Vol. 2, pp 1–758. New York: Springer.

[B28b] Haufe S, Meinecke F, Görgen K, Dähne S, Haynes JD, Blankertz B, Bießmann F (2014) On the interpretation of weight vectors of linear models in multivariate neuroimaging. Neuroimage 87:96–110. 10.1016/j.neuroimage.2013.10.067 24239590

[B29] Heusser AC, Ezzyat Y, Shiff I, Davachi L (2018) Perceptual boundaries cause mnemonic trade-offs between local boundary processing and across-trial associative binding. J Exp Psychol Learn Mem Cogn 44:1075. 10.1037/xlm0000503 29461067 PMC6013306

[B30] Hindy NC, Ng FY, Turk-Browne NB (2016) Linking pattern completion in the hippocampus to predictive coding in visual cortex. Nat Neurosci 19:665–667. 10.1038/nn.4284 27065363 PMC4948994

[B31] Hutchinson JB, Barrett LF (2019) The power of predictions: an emerging paradigm for psychological research. Curr Dir Psychol Sci 28:280–291. 10.1177/0963721419831992 31749520 PMC6867616

[B31a] Izenman AJ (2008) Modern multivariate statistical techniques. Vol. 1. New York: Springer.

[B32] Keidel JL, Oedekoven CS, Tut AC, Bird CM (2018) Multiscale integration of contextual information during a naturalistic task. Cereb Cortex 28:3531–3539. 10.1093/cercor/bhx218 28968727

[B33] Kim G, Norman KA, Turk-Browne NB (2017) Neural differentiation of incorrectly predicted memories. J Neurosci 37:2022–2031. 10.1523/JNEUROSCI.3272-16.2017 28115478 PMC5338753

[B34] Klimesch W (1999) EEG alpha and theta oscillations reflect cognitive and memory performance: a review and analysis. Brain Res Rev 29:169–195. 10.1016/S0165-0173(98)00056-3 10209231

[B35] Knight RT, Scabini D, Woods DL, Clayworth CC (1989) Contributions of temporal-parietal junction to the human auditory P3. Brain Res 502:109–116. 10.1016/0006-8993(89)90466-6 2819449

[B36] Kurby CA, Zacks JM (2008) Segmentation in the perception and memory of events. Trends Cogn Sci 12:72–79. 10.1016/j.tics.2007.11.004 18178125 PMC2263140

[B37] Lee CS, Aly M, Baldassano C (2021) Anticipation of temporally structured events in the brain. eLife 10:e64972. 10.7554/eLife.64972 33884953 PMC8169103

[B38] Lohnas LJ, Healey MK, Davachi L (2023) Neural temporal context reinstatement of event structure during memory recall. J Exp Psychol Gen 152:1840–1872. 10.1037/xge0001354 37036669 PMC10293072

[B39] Long NM, Burke JF, Kahana MJ (2014) Subsequent memory effect in intracranial and scalp EEG. Neuroimage 84:488–494. 10.1016/j.neuroimage.2013.08.052 24012858 PMC3849113

[B40] Long NM, Kuhl BA (2019) Decoding the tradeoff between encoding and retrieval to predict memory for overlapping events. Neuroimage 201:116001. 10.1016/j.neuroimage.2019.07.014 31299369 PMC6765409

[B41] Lositsky O, Chen J, Toker D, Honey CJ, Shvartsman M, Poppenk JL, Hasson U, Norman KA (2016) Neural pattern change during encoding of a narrative predicts retrospective duration estimates. eLife 5:e16070. 10.7554/eLife.16070 27801645 PMC5243117

[B42] Luck SJ (2014) An introduction to the event-related potential technique. Cambridge, MA: MIT press.

[B42a] Lu Q, Hasson U, Norman KA (2022) A neural network model of when to retrieve and encode episodic memories. Elife 11:e74445. 10.7554/eLife.74445 35142289 PMC9000961

[B43] Mack ML, Preston AR, Love BC (2020) Ventromedial prefrontal cortex compression during concept learning. Nat Commun 11:46. 10.1038/s41467-019-13930-8 31911628 PMC6946809

[B44] Manning JR, Polyn SM, Baltuch GH, Litt B, Kahana MJ (2011) Oscillatory patterns in temporal lobe reveal context reinstatement during memory search. Proc Natl Acad Sci U S A 108:12893–12897. 10.1073/pnas.1015174108 21737744 PMC3150951

[B45] Mather M, Clewett D, Sakaki M, Harley CW (2016) Norepinephrine ignites local hotspots of neuronal excitation: how arousal amplifies selectivity in perception and memory. Behav Brain Sci 39:e200. 10.1017/S0140525X15000667 26126507 PMC5830137

[B46] Michelmann S, et al. (2021) Moment-by-moment tracking of naturalistic learning and its underlying hippocampo-cortical interactions. Nat Commun 12:5394. 10.1038/s41467-021-25376-y 34518520 PMC8438040

[B46a] Momennejad I, Russek EM, Cheong JH, Botvinick MM, Daw ND, Gershman SJ (2017) The successor representation in human reinforcement learning. Nat Hum Behav 1:680–692. 10.1038/s41562-017-0180-8 31024137 PMC6941356

[B47] Morton NW, Schlichting ML, Preston AR (2020) Representations of common event structure in medial temporal lobe and frontoparietal cortex support efficient inference. Proc Natl Acad Sci U S A 117:29338–29345. 10.1073/pnas.1912338117 33229532 PMC7703583

[B48] Nakano T, Yamamoto Y, Kitajo K, Takahashi T, Kitazawa S (2009) Synchronization of spontaneous eyeblinks while viewing video stories. Proc R Soc B Biol Sci 276:3635–3644. 10.1098/rspb.2009.0828 19640888 PMC2817301

[B49] Newtson D (1973) Attribution and the unit of perception of ongoing behavior. J Pers Soc Psychol 28:28–38. 10.1037/h0035584

[B50] Nieuwenhuis S, Aston-Jones G, Cohen JD (2005) Decision making, the P3, and the locus coeruleus–norepinephrine system. Psychol Bull 131:510. 10.1037/0033-2909.131.4.510 16060800

[B51] Pineda JA, Foote SL, Neville HJ (1989) Effects of locus coeruleus lesions on auditory, long-latency, event-related potentials in monkey. J Neurosci 9:81–93. 10.1523/JNEUROSCI.09-01-00081.1989 2563282 PMC6570008

[B52] Poe GR, et al. (2020) Locus coeruleus: a new look at the blue spot. Nat Rev Neurosci 21:644–659. 10.1038/s41583-020-0360-9 32943779 PMC8991985

[B53] Polich J (2012) Neuropsychology of P300. In: The Oxford handbook of event-related potential components (Luck S, Kappenman E, eds), pp 159–188. New York, NY: Oxford University Press.

[B54] Polich J, Kok A (1995) Cognitive and biological determinants of P300: an integrative review. Biol Psychol 41:103–146. 10.1016/0301-0511(95)05130-9 8534788

[B55] Polyn SM, Norman KA, Kahana MJ (2009) Task context and organization in free recall. Neuropsychologia 47:2158–2163. 10.1016/j.neuropsychologia.2009.02.013 19524086 PMC2697131

[B56] Press C, Kok P, Yon D (2020) The perceptual prediction paradox. Trends Cogn Sci 24:13–24. 10.1016/j.tics.2019.11.003 31787500

[B56a] Pu Y, Kong XZ, Ranganath C, Melloni L (2022) Event boundaries shape temporal organization of memory by resetting temporal context. Nat Commun 13:622. 10.1038/s41467-022-28216-9 35110527 PMC8810807

[B57] Pudhiyidath A, Morton NW, Viveros Duran R, Schapiro AC, Momennejad I, Hinojosa- Rowland DM, Molitor RJ, Preston AR (2022) Representations of temporal community structure in hippocampus and precuneus predict inductive reasoning decisions. J Cogn Neurosci 34:1736–1760. 10.1162/jocn_a_01864 35579986 PMC10262802

[B58] Radvansky GA, Copeland DE (2006) Walking through doorways causes forgetting: situation models and experienced space. Mem Cognit 34:1150–1156. 10.3758/BF03193261 17128613

[B59] Rao RP, Ballard DH (1999) Predictive coding in the visual cortex: a functional interpretation of some extra-classical receptive-field effects. Nat Neurosci 2:79–87. 10.1038/4580 10195184

[B60] Reynolds JR, Zacks JM, Braver TS (2007) A computational model of event segmentation from perceptual prediction. Cogn Sci 31:613–643. 10.1080/15326900701399913 21635310

[B61] Richmond LL, Zacks JM (2017) Constructing experience: event models from perception to action. Trends Cogn Sci 21:962–980. 10.1016/j.tics.2017.08.005 28899609 PMC5694361

[B62] Rouhani N, Norman KA, Niv Y (2018) Dissociable effects of surprising rewards on learning and memory. J Exp Psychol Learn Mem Cogn 44:1430. 10.1037/xlm0000518 29553767 PMC6117220

[B62a] Rouhani N, Norman KA, Niv Y, Bornstein AM (2020) Reward prediction errors create event boundaries in memory. Cognition 203:104269. 10.1016/j.cognition.2020.104269 32563083 PMC7483902

[B63] Saffran JR, Johnson EK, Aslin RN, Newport EL (1999) Statistical learning of tone sequences by human infants and adults. Cognition 70:27–52. 10.1016/S0010-0277(98)00075-4 10193055

[B64] Sara SJ (2009) The locus coeruleus and noradrenergic modulation of cognition. Nat Rev Neurosci 10:211–223. 10.1038/nrn2573 19190638

[B65] Sara SJ (2015) Locus coeruleus in time with the making of memories. Curr Opin Neurobiol 35:87–94. 10.1016/j.conb.2015.07.004 26241632

[B66] Sauseng P, Griesmayr B, Freunberger R, Klimesch W (2010) Control mechanisms in working memory: a possible function of EEG theta oscillations. Neurosci Biobehav Rev 34:1015–1022. 10.1016/j.neubiorev.2009.12.006 20006645

[B67] Schapiro AC, Kustner LV, Turk-Browne NB (2012) Shaping of object representations in the human medial temporal lobe based on temporal regularities. Curr Biol 22:1622–1627. 10.1016/j.cub.2012.06.056 22885059 PMC3443305

[B68] Schapiro AC, Rogers TT, Cordova NI, Turk-Browne NB, Botvinick MM (2013) Neural representations of events arise from temporal community structure. Nat Neurosci 16:486–492. 10.1038/nn.3331 23416451 PMC3749823

[B69] Schapiro AC, Turk-Browne NB, Norman KA, Botvinick MM (2016) Statistical learning of temporal community structure in the hippocampus. Hippocampus 26:3–8. 10.1002/hipo.22523 26332666 PMC4715493

[B70] Schlichting ML, Preston AR (2015) Memory integration: neural mechanisms and implications for behavior. Curr Opin Behav Sci 1:1–8. 10.1016/j.cobeha.2014.07.005 25750931 PMC4346341

[B71] Shannon CE (1948) A mathematical theory of communication. Bell Syst Tech J 27:379–423. 10.1002/j.1538-7305.1948.tb01338.x

[B72] Sherman BE, DuBrow S, Winawer J, Davachi L (2023) Mnemonic content and hippocampal patterns shape judgments of time. Psychol Sci 34:221–237. 10.1177/09567976221129533 36442582 PMC10068509

[B73] Sherman BE, Turk-Browne NB (2020) Statistical prediction of the future impairs episodic encoding of the present. Proc Natl Acad Sci U S A 117:22760–22770. 10.1073/pnas.2013291117 32859755 PMC7502714

[B73a] Sherman BE, Graves KN, Turk-Browne NB (2020) The prevalence and importance of statistical learning in human cognition and behavior. Curr Opin Behav Sci 32:15–20. 10.1016/j.cobeha.2020.01.015 32258249 PMC7108790

[B74] Shin YS, DuBrow S (2021) Structuring memory through inference-based event segmentation. Top Cogn Sci 13:106–127. 10.1111/tops.12505 32459391

[B75] Siefke BM, Smith TA, Sederberg PB (2019) A context-change account of temporal distinctiveness. Mem Cognit 47:1158–1172. 10.3758/s13421-019-00925-5 30912034

[B76] Silva M, Baldassano C, Fuentemilla L (2019) Rapid memory reactivation at movie event boundaries promotes episodic encoding. J Neurosci 39:8538–8548. 10.1523/JNEUROSCI.0360-19.2019 31519818 PMC6807272

[B77] Simony E, Honey CJ, Chen J, Lositsky O, Yeshurun Y, Wiesel A, Hasson U (2016) Dynamic reconfiguration of the default mode network during narrative comprehension. Nat Commun 7:12141. 10.1038/ncomms12141 27424918 PMC4960303

[B78] Sinclair AH, Barense MD (2018) Surprise and destabilize: prediction error influences episodic memory reconsolidation. Learn Mem 25:369. 10.1101/lm.046912.117 30012882 PMC6049395

[B79] Smith DE, Moore IL, Long NM (2022) Temporal context modulates encoding and retrieval of overlapping events. J Neurosci 42:3000–3010. 10.1523/JNEUROSCI.1091-21.2022 35232765 PMC8985871

[B80] Sols I, DuBrow S, Davachi L, Fuentemilla L (2017) Event boundaries trigger rapid memory reinstatement of the prior events to promote their representation in long-term memory. Curr Biol 27:3499–3504. 10.1016/j.cub.2017.09.057 29129536 PMC6398599

[B81] Speer NK, Zacks JM (2005) Temporal changes as event boundaries: processing and memory consequences of narrative time shifts. J Mem Lang 53:125–140. 10.1016/j.jml.2005.02.009

[B82] Staresina BP, Fell J, Do Lam AT, Axmacher N, Henson RN (2012) Memory signals are temporally dissociated in and across human hippocampus and perirhinal cortex. Nat Neurosci 15:1167–1173. 10.1038/nn.3154 22751037 PMC3428860

[B83] Stawarczyk D, Bezdek MA, Zacks JM (2021) Event representations and predictive processing: the role of the midline default network core. Top Cogn Sci 13:164–186. 10.1111/tops.12450 31486286 PMC7984453

[B84] Swallow KM, Broitman AW, Riley E, Turker HB (2022) Grounding the attentional boost effect in events and the efficient brain. Front Psychol 13:892416. 10.3389/fpsyg.2022.892416 35936250 PMC9355572

[B85] Swallow KM, Zacks JM, Abrams RA (2009) Event boundaries in perception affect memory encoding and updating. J Exp Psychol Gen 138:236. 10.1037/a0015631 19397382 PMC2819197

[B86] Turker HB, Swallow KM (2019) Attending to behaviorally relevant moments enhances incidental relational memory. Mem Cognit 47:1–16. 10.3758/s13421-018-0846-0 30097907

[B86a] Verleger R (1998) Toward an integration of P3 research with cognitive neuroscience. *Behavioral and Brain Sciences*, 21:150–152. 10.1017/S0140525X98220954

[B87] Verleger R, Heide W, Butt C, Kömpf D (1994) Reduction of P3b in patients with temporo-parietal lesions. Cogn Brain Res 2:103–116. 10.1016/0926-6410(94)90007-8 7833690

[B88] von Stein A, Sarnthein J (2000) EEG frequency and the size of cognitive neuronal assemblies. Cogn Brain Res 23:413–414. 10.1017/S0140525X0038325X

[B88a] Weidemann CT, Mollison MV, Kahana MJ (2009) Electrophysiological correlates of high-level perception during spatial navigation. Psychon Bull Rev 16:313–319. 10.3758/PBR.16.2.313 19293100 PMC2704578

[B89] Weidemann CT, et al. (2019) Neural activity reveals interactions between episodic and semantic memory systems during retrieval. J Exp Psychol Gen 148:1. 10.1037/xge0000480 30596439 PMC6419095

[B90] Yamaguchi S, Knight RT (1991) Anterior and posterior association cortex contributions to the somatosensory P300. J Neurosci 11:2039–2054. 10.1523/JNEUROSCI.11-07-02039.1991 2066773 PMC6575477

[B91] Yu AJ, Dayan P (2005) Uncertainty, neuromodulation, and attention. Neuron 46:681–692. 10.1016/j.neuron.2005.04.026 15944135

[B92] Zacks JM, Speer NK, Swallow KM, Braver TS, Reynolds JR (2007) Event perception: a mind-brain perspective. Psychol Bull 133:273. 10.1037/0033-2909.133.2.273 17338600 PMC2852534

[B93] Zacks JM, Tversky B (2001) Event structure in perception and conception. Psychol Bull 127:3. 10.1037/0033-2909.127.1.3 11271755

[B93a] Zacks JM, Kurby CA, Eisenberg ML, Haroutunian N (2011) Prediction error associated with the perceptual segmentation of naturalistic events. J Cogn Neurosci 23:4057–4066. 10.1162/jocn_a_00078 21671745 PMC8653780

[B94] Zeithamova D, Bowman CR (2020) Generalization and the hippocampus: more than one story? Neurobiol Learn Mem 175:107317. 10.1016/j.nlm.2020.107317 33007461 PMC7655622

[B95] Zheng J, Schjetnan AG, Yebra M, Gomes BA, Mosher CP, Kalia SK, Valiante TA, Mamelak AN, Kreiman G, Rutishauser U (2022) Neurons detect cognitive boundaries to structure episodic memories in humans. Nat Neurosci 25:358–368. 10.1038/s41593-022-01020-w 35260859 PMC8966433

[B96] Zwaan RA (1996) Processing narrative time shifts. J Exp Psychol Learn Mem Cogn 22:1196. 10.1037/0278-7393.22.5.1196

